# Editorial: Ion Channels and Transporters in Ca^2+^ -Dependent Functions of Lymphocytes

**DOI:** 10.3389/fphys.2022.962110

**Published:** 2022-07-07

**Authors:** Peter Hajdu, Heike Wulff, Barbara A. Niemeyer, Ildikó Szabó

**Affiliations:** ^1^ Department of Biophysics and Cell Biology, University of Debrecen, Debrecen, Hungary; ^2^ Department of Dental Biochemistry, Faculty of Dentistry, University of Debrecen, Debrecen, Hungary; ^3^ Department of Pharmacology, School of Medicine, University of California, Davis, CA, Davis, United States; ^4^ Department of Molecular Biophysics, Faculty of Medicine, Saarland University, Homburg, Germany; ^5^ Department of Biology, Faculty of Sciences, University of Padua, Padua, Italy

**Keywords:** ion channels, ion transporter, lymhocyte, Ca^2+^- signaling, cancer, autoimmunity, pharmacology

Ion channels and transporters have been shown to play essential roles in the physiology of lymphocytes since their discovery in the 1980s. Understanding the specific interplay between lymphocyte ion channels and the particular roles of defined ion channels and transporters for immune cells and subtype specific functions is crucial for untangling the pathophysiological mechanisms behind numerous immune system related diseases and may uncover new targets for pharmaceutical intervention.

This special topic, which is jointly issued in Frontiers in Physiology and Frontiers in Pharmacology, contains 8 original research studies and one review article. The articles cover topics ranging from classical pharmacology of ion channels, the effects of excess intracellular Ca^2+^ induced by exercise to the role/expression of various ion channels under pathological conditions, and it is noteworthy, that the included articles report on almost all major ion channels/transporter described in lymphocytes.

Two articles in our collection focus on the role of ion channels and expression level changes in disease. In their study Markakis et al. show increases in functional Kv1.3 expression in T cells from multiple sclerosis (MS) patients, which is more pronounced in patients with secondary progressive MS status (Markakis et al.). Furthermore, the authors also reveal that TASK-2 channels have no influence on the increased K^+^ conductance detected in MS patients. In the second article by Gawali et al., we learn about the effect of immune checkpoint therapies on K+ channel function in CD8^+^ cells: treatment with both anti-PD1 (pembrolizumab) and anti-PD-L1 (atezolizumab) antibodies facilitate the function of KCa3.1 and Kv1.3 in head and neck squamous cell carcinoma patients’ peripheral lymphocytes (Gawali et al.). Treatment of CD8^+^ cells isolated from healthy donors with PD-L1 reduced KCa3.1 activity, an effect that could be attributed to the modulation by PI3 kinase. However, addition of anti-PD1 did not change the expression level of Kv1.3, KCa3.1, and Orai1/STIM1 as determined by flow cytometry.

Three articles report on the pharmacology of lymphocyte ion channels, thus providing insight into therapy development or explaining side effects of clinically used drugs. Loureirin B (LrB), which is a flavonoid extracted from Resina Draconis (“dragon’s blood”), was shown to inhibit Kv1.3 channels as well as CRAC channels constituted of STIM1/Orai1 complexes in a concentration-dependent manner (Shi et al.). Moreover, LrB reduces Ca^2+^ influx and IL-2 production in Jurkat T cells. Hence, loureirin B may serve as a template for developing new small molecule inhibitors to treat autoimmune diseases. In a second article, several broadly-used NSAID compounds (naproxen, ibuprofen, salicylate, and aspirin) are shown to inhibit TRPM7 currents (Chokshi et al.). It turned out that these molecules develop use-dependent block slowly and reduce cell viability in a concentration dependent manner, however, they do not bind directly to TRPM7 channels but impair TRPM7 function via acidification of the cytosolic compartment. A specific mutation within TRPM7 (S1170R) renders the channel resistant to intracellular pH changes, Mg^2+^ and PIP_2_ and, indeed, is resistant to NSAID application. The pH-change induced inhibition did not require activity of cyclooxygenase enzymes, another well-known target of NSAIDs. In the final article of this set, Naseem et al. describe a new expression system suitable for production of peptide toxins with a high yield and purity (three-fold higher as compared to other systems) (Naseem et al.). As these toxins often are very specific inhibitors of subtype specific ion channels, the more technical paper is of interest to scientists studying the role of ion channels for immune cell pathologies. The authors chose margatoxin (MgTx), which is a high affinity antagonist of Kv1.2 and Kv1.3 and produced the toxin in *Pichia pastoris* (a methylotrophic yeast species) in a tagged (TrMgTx, with 6xHis tag) and an untagged (UrMgTx) version. Both variants had an inhibitory effect comparable to wild-type MgTx, and they could show that toxins were able to inhibit the expression of early activation markers (IL2 receptor or CD25 and CD40L or CD154) in CD4^+^ effector memory T cells. The *Pichia pastoris* expression system proves to be efficient tool in generating cysteine rich small peptides such as recombinant toxins applied in study of ion channels function and expression.

Another set of three articles was devoted to aspects of Ca^2+^ signaling in T cells. Much recent interest focusses on the role of mitochondrial Ca^2+^ handling. Wu et al. report on the role of the mitochondrial Ca^2+^ uniporter (MCU), which is one pathway for Ca^2+^ uptake into mitochondria for various T cell functions (Wu et al.). Using mice with T cell-specific genetic elimination of MCU, the authors conclude that lack of MCU has no influence on the respiratory chain activity, T cell differentiation or effector functions in both regulatory and inflammatory T cell populations *in vitro*. *In vivo,* mouse model experiments demonstrate that MCU also does not appear to play a significant role: not for the development of T-cell mediated autoimmunity and not for immune responses against viral infections. These results draw attention to the fact that compensatory mechanisms may override effects of genetic ablation of MCU as well as suggest that antagonists used in other studies might not be specific towards MCU. In the paper by Liu et al. the effects of exercise (running in/on treadmill) on Ca^2+^ levels in T cells was reported: basal resting cytosolic Ca^2+^-concentrations as well as agonist-induced Ca^2+^-responses in cells isolated 3 h (E3 group) after workout were higher as compared to the 0 h (E0 group) and 24 h (E24 group) groups. In contrast, proliferation rates in the E3 group were lower relative to the other groups (Liu et al.). Transcriptional levels of SERCA, PMCA, TRPC1 and P2X7 are downregulated in E3 T cells, while IP_3_R2 and RyR2 expression levels are up-regulated: these changes could account for the increased cytosolic Ca^2+^ level of E3 T cells. However, the suppressed, mitogen-induced proliferation of E3 cells contradicts data obtained for Ca^2+^ levels in the E3 group cells and suggests that different pathway are responsible for exercise-related immune-suppression. Finally, a study on various lymphoma cell lines and primary lymphoma cells (DLBCL: diffuse large B cell lymphoma) analyzed expression of several Ca_V_ channel genes as well as the Orai-STIM-ORAI gene set (Stanwood et al.). Cav1.2 expression was higher in classical Hodgkin lymphoma (CHL) cell lines as compared to other B cell lymphoma cell lines, while STIM2-Orai2-levels were decreased. Activated B cell like DLBCL (ABC-DLBCL) cells had higher levels of Ca_V_1.3, while Ca_V_1.1, Ca_V_1.2 and Ca_V_1.4 were reduced in comparison to germinal center DLCBL cells, and no difference in STIM-ORAI expression was reported. Other proteins related to the Ca^2+^-regulated activation pathway (NFAT, calcineurin, calmodulin) displayed elevated expression in CHL cell lines with respect to other B lymphoma cell lines. In contrast, the calmodulin and Ca^2+^-dependent pathway was downregulated in ABC-DLBCL patient cells. A recent study reported that Ca_V_ channel transcripts lacking huge parts of 5’ exons were present in T cells, which probably transcribes their mRNA into non-functional truncated Ca_V_ proteins: no Ca^2+^ channels were detected by electrophysiology tools (patch–clamp, Ca^2+^-imaging) (Erdogmus et al., 2022). The situation may be the same for B cells, hence, functional studies are required to clarify role of Ca_V_ in B lymphocytes and lymphoma cells.

Last, but not least, a review by Bohmwald et al. described the role of Ca^2+^-signaling in viral infection and showcases how viruses highjack host cell Ca^2+^ channels to facilitate both their entry into cells as well as their replication and budding capacity (Bohmwald et al.). The authors summarize the data on ion channels expressed in B and T lymphocytes, and selected viruses (Hepatitis B, SARS-CoV-2, Herpes simplex, hRSV, and HIV), all of which utilize Ca^2+^-signaling pathways by modifying Ca^2+^ channel function in order to enter cells and/or replicate.

Taken together, this article collection highlights the importance of ion channels and transporters regulating calcium signaling in lymphocytes in health and disease.
